# Perinatal depression screening: a systematic review of recommendations from member countries of the Organisation for Economic Co-operation and Development (OECD)

**DOI:** 10.1007/s00737-022-01249-1

**Published:** 2022-07-18

**Authors:** Sarira El-Den, Lily Pham, Isobel Anderson, Shan Yang, Rebekah J. Moles, Claire L. O’Reilly, Philip Boyce, Karen Hazell Raine, Camille Raynes-Greenow

**Affiliations:** 1 The University of Sydney School of Pharmacy, Faculty of Medicine and Health, The University of Sydney, Sydney, NSW 2006 Australia; 2 The University of Sydney School of Public Health, Faculty of Medicine and Health, The University of Sydney, Sydney, NSW 2006 Australia; 3 The University of Sydney School of Medicine, Faculty of Medicine and Health, The University of Sydney, Sydney, NSW 2006 Australia; 4 The Westmead Institute for Medical Research, Sydney, NSW 2145 Australia; 5 The University of Sydney School of Nursing, Faculty of Medicine and Health, The University of Sydney, Sydney, NSW 2006 Australia; 6 College of Nursing and Midwifery, Charles Darwin University, Darwin, NT 0909 Australia

**Keywords:** Perinatal mental health, Postpartum care, Preventive health care, Screening and diagnostic tests

## Abstract

Perinatal depression (PND) screening recommendations are made by national, state-based and professional organisations; however, there is disagreement regarding screening timing, provider responsible, screening setting, screening tool as well as the follow-up and referral pathways required post-screening. This systematic review aimed to identify, describe and compare PND screening recommendations from member countries of the Organisation for Economic Co-operation and Development (OECD). Publications were identified through systematically searching PubMed, Google and the Guidelines International Network (GIN). Recommendations regarding PND screening endorsement, timing, frequency, responsible provider, tools/assessments and follow-up and referral were extracted. Twenty-one publications, including guidelines, from five countries were included. Most made recommendations in support of PND screening using the Edinburgh Postnatal Depression Scale. Details differed regarding terminology used, as well as frequency of screening, follow-up mechanisms and referral pathways. A broad range of health providers were considered to be responsible for screening. This is the first review to identify and compare PND screening recommendations from OECD member countries; however, only online publications published in English, from five countries were included. Heterogeneity of publication types and inconsistency in definitions rendered quality assessment inappropriate. While most publications generally endorsed PND screening, there are exceptions and the associated details pertaining to the actual conduct of screening vary between and within countries. Developing clear, standardised recommendations based on current evidence is necessary to ensure clarity amongst healthcare providers and a comprehensive approach for the early detection of PND.

## Introduction

Perinatal depression (PND) — an episode of depression occurring during pregnancy or the first postpartum year (Bauer et al. 2014) — affects approximately 10–20% of women globally (AIHW 2012; Selix & Goyal 2018; World Health Organization 2021). There is a lack of consistency across official diagnostic criteria when defining PND, especially regarding the postnatal period (Howard & Khalifeh 2020). The DSM-5 uses a “peripartum” specifier when the onset of a depressive disorder occurs during pregnancy, or in the 4 weeks following delivery (American Psychiatric Association 2013), while the International Classification of Diseases 11th Revision (ICD-11) defines the onset of a mental disorder in the puerperium as that which “commences within about 6 weeks after delivery” (World Health Organisation 2020). These “official” diagnoses often differ significantly to clinical opinion and longitudinal studies demonstrating that women are at risk of developing mental health problems up to 5 months postpartum (Munk-Olsen et al., 2006), and published literature often alludes to the postnatal period as up to 12 months postpartum (El-Den et al. 2015; Selix & Goyal 2018). The lack of consensus as to what constitutes the postpartum period may contribute to variability in PND screening recommendations and practices.

PND often goes unidentified with many women not recognizing that their symptoms could be indicative of a mental illness, resulting in a lack of diagnosis and treatment (Cox et al. 2016). Screening has been proposed to allow for earlier identification and appropriate referral for diagnosis and treatment.

PND is associated with poorer health outcomes for both the mother and the baby (Stein et al. 2014), such as an increased risk of low birthweight and preterm birth antenatally (Dadi et al., 2020) and adverse infant developmental outcomes postnatally (O'Hara 2009). There are effective, evidence-based treatment options for PND including psychotherapy and antidepressant medication (O'Connor et al. 2016). Therefore, early identification may allow for timely management and treatment, thereby potentially reducing the risk of these adverse outcomes (Pearlstein et al. 2009). Screening for postnatal depression has been evaluated, with evidence to demonstrate reduced depression risk among women who participate in screening programs (O'Connor et al. 2016). Furthermore, PND screening can lead to increased rates of referrals and service use (Reilly et al. 2020).

Nonetheless, there is debate in the literature as to whether PND screening should be provided routinely (AE Buist et al. 2002; Hazell Raine et al. 2020), due to a lack of evidence demonstrating the effectiveness of screening in improving patient outcomes (Thombs et al. 2014). A systematic review published in 2014 reported that the evidence did not demonstrate effectiveness of PND screening and that robust randomized controlled trials (RCTs) exploring effectiveness and cost-effectiveness are necessary (Thombs et al. 2014). Furthermore, it is important to note that screening for PND, specifically using screening tools such as the Edinburgh Postnatal Depression Scale (EPDS) does not allow for a diagnosis of a depressive disorder, nor does it allow for the identification of women experiencing other mental illnesses, which may be less common, such as personality disorders, bipolar disorder and postpartum psychosis (Boyce & Judd 2019; Judd et al. 2017). Rather, screening, using tools such as the EPDS, can identify those who require further assessment for diagnosis of depression and/or anxiety. Moreover, the psychometric properties of the screening tool also need to be considered in relation to the illness for which it is being used (AE Buist et al. 2002). For example, the EPDS has been used to screen for both anxiety and depressive disorders (Matthey 2008). A screening tool with poor reliability and validity can lead to false positives and ultimately overdiagnosis, as well as false negatives which can be costly (Thombs et al. 2014). Reviews exploring the psychometric properties of depression screening tools in primary care settings, generally (S. El-Den et al. 2018a) and specifically in relation to PND, demonstrate that the EPDS and the Patient Health Questionniare-9 (PHQ-9) are valid and reliable screening tools across various settings when used by a range of trained healthcare professionals (Levis et al. 2020; Wang et al. 2021).

Concerns surrounding screening also often include debate as to whether routine screening is acceptable (AE Buist et al. 2002); however, a systematic review exploring acceptability among key stakeholders, demonstrated that screening is generally acceptable to perinatal women and health professionals (El-Den et al. 2015). There is a growing body of evidence relating to PND screening by a range of health care professionals, such as physicians (Ford et al. 2017), nurses (Segre et al. 2010), midwives (Martin et al. 2020), paediatricians (Byatt et al. 2013; Chambers et al. 2019; Currie & Rademacher 2004) and pharmacists (El-Den et al. 2019; Sarira El-Den et al. 2018a, b, c; Elkhodr et al. 2018); hence, it is often unclear which provider is responsible for screening and there is growing support for integrating early detection in all “medical settings that encounter perinatal women” (Flynn et al. 2006). If screening is to be delivered in a variety of medical settings, then in addition to ensuring healthcare professionals working in those settings are trained, there is a need to also ensure that appropriate, site-specific follow-up and referral pathways for diagnostic assessment and treatment are also established.

Considering the lack of consensus relating to PND screening, the aim of the current review was to identify and compare recommendations relating to PND screening across countries that are members of the Organisation for Economic Co-operation and Development (OECD).

## Methods

This systematic review was guided by the PRISMA statement (Moher et al. 2009; Page et al. 2021).

### Search strategy

Recommendations pertaining to PND screening from the 37 countries that were members of the OECD in September 2020 (OECD 2020) were identified by searching PubMed, the World Wide Web via Google search engine and the Guidelines International Network (GIN) library. The detailed search strategy for each search is presented in Table 1. The searches were conducted in September 2020, and no date restrictions were applied.

**Table 1:** Search strategy

Database/ search engine	PubMed	World Wide Web was searched via Google search engine	Guidelines International Network (GIN) library
Terms	(*Perinatal OR postnatal OR pregnancy OR postpartum OR maternal OR antenatal*); *AND Depression; AND Screening; NOT Cancer*	(*Guidelines OR recommendations*) *AND* (*screening OR risk assessment OR early detection*) *AND* (*Major depressive disorder OR depression*) *AND* (*postnatal OR maternal OR peripartum OR postpartum OR perinatal OR antenatal OR pregnancy*) *AND “country name.”*	Three keyword searches: 1. “*mental health*” 2. “*depression*” 3. “*perinatal*”
Filters	“Guidelines”	N/A	N/A

The Google search was repeated for each of the 37 OECD member countries, whereby for each search, the URLs, country of origin, publishing organisation/journal and document/page title for the first 40 results were extracted and exported to an Excel spreadsheet.

While screening and extracting data from full-text citations (e.g. online documents, webpages), if in-text reference to a potentially relevant publication was identified, co-authors attempted to find it and screen it as well.

### Inclusion and exclusion criteria

Publications from all OECD member countries, regardless of the issuing organisation (e.g. body, college, committee, institution, association, society, department), were eligible for inclusion, provided that they were published online (at the time of searching and screening), and it was clear (e.g. on the title page) that the recommendation was published by or on behalf of an organisation within an OECD member country, or specifically stipulated that it was intended for adoption within an OECD member country. Provided these criteria were met, a publication was considered eligible for inclusion if it made a recommendation relating to screening for depression and/or risk factors for depression during the perinatal period. Terms such as “screening”, “risk assessment”, “early detection”, “enquiry” and “identification” are often used interchangeably in the literature to refer to preventative health care which “aims to reduce the burden of chronic conditions by early identification of people with risk factors or symptoms and applying appropriate interventions” and psychosocial assessment can encompass depression screening (Austin 2014). Hence, if a publication did not mention the term “screening”, specifically, but it could be ascertained that the publication was making a recommendation pertaining to assessing for, asking about or enquiring about depressive symptoms or risk of depression during the perinatal period for the purpose of identification and “applying appropriate interventions” (e.g. referral/follow-up for diagnostic assessment), then it was eligible for inclusion (Austin 2014). A broad range of publication types were eligible for inclusion, such as guidelines, guidance, recommendations, reports, statements (e.g. position statement, policy statement), consultations, opinions (e.g. committee opinion, organisation opinion) and health professional resources (e.g. toolkit, manual, guide, handbook, booklet).

Publications, such as journal articles, whereby the key focus appeared to be to report on primary research studies only, evaluate evidence or conduct a literature review, as well as publications that were abstracts only, were ineligible for inclusion. Publications were also excluded if written in a language other than English. There was no limit on publication date; however, if there were different versions of the same publication available, only the current/most recent publication was included. Publications that only endorsed pre-existing PND screening recommendations (e.g. by other organisations) and did not develop or make their own recommendation were also excluded.

### Study selection and data extraction

Publications identified were exported into Excel, where duplicates were removed. Two authors (IA and SY) then screened the titles, abstracts and full-texts for inclusion and discussed any disagreements to reach consensus. Where disagreements could not be resolved, the publications were reviewed by two additional authors (SED and CRG) to reach consensus. During full-text screening and data extraction, if in-text references to other PND screening recommendations were identified, then potentially relevant references were also screened for inclusion.

Data extraction was led by LP and focused on the PND or PND risk screening recommendation, provider responsible, screening tool recommended, screening frequency and follow-up and referral pathways recommended (Table 2). Every attempt was made to extract data that pertained to depression and the perinatal period specifically, especially for publications whereby the scope of the publication was broader than depression (e.g. mental illness) or the perinatal period (e.g. adults). This was to ensure extracted data was relevant to the aims of this review.

**Table 2:** Summary of recommendations relating to perinatal depression screening

	Document name and publication year	Organisation and governance level	Publication type	Period	Recommendation	Key provider responsible	Tool	Frequency and timing	Follow-up and referral
AUSTRALIA									
1	SAFE START Guidelines: Improving mental health outcomes for parents and infants 2010	Department of Health, NSW *State*	Guideline	Perinatal	A range of bio-psychosocial factors that can contribute to health problems and disorders for mothers and infants is captured in the psychosocial assessment questions developed by NSW Health and recommended for *universal* use in NSW in antenatal and postnatal maternity and child & family health settings Psychosocial assessment includes depression *screening*. The psychosocial assessment should be incorporated as part of a comprehensive assessment for *all* women during pregnancy and again after the baby is born	Clinician (midwife, child and family health nurse)	SAFE START Psychosocial assessment which includes EDS/EPDS	The EDS/EPDS complements the psychosocial assessment questions administered routinely at the first opportunity during pregnancy and repeated in the postnatal period	If past/ongoing history of mental disorders and personality vulnerability is identified then respond with a comprehensive mental health assessment (includes diagnosis, history, current or past treating mental health team/psychiatrist, medications, as well as a comprehensive plan to enhance coping skills and lower vulnerability during the birth and the postnatal period) Key services: Mental health – private and public sector, GPs Sometimes families may be reluctant to acknowledge this or seek out treatment. For this reason, if current symptoms of mental health problems are identified, non-threatening, assertive, home-based, follow-up care is recommended. For women experiencing severe clinical symptoms during the perinatal period, three levels of intervention include psychiatrist/GP, Community Adult Mental Health Services and/or Hospitalisation
2	Perinatal Anxiety and Depression 2015	RANZCOG *National*	Guideline	Perinatal	*Screening* for perinatal mood disorders should be considered part of *routine* antenatal and postpartum care	Clinician, practitioner	Validated tool (e.g., EPDS)	*Routine* antenatal and postpartum screening	Managing a mental health problem should be collaborative. Referral requires consent from the mother, and referral options and/ or treatment plan should take into account the mother’s preferences. In most cases, referral will be to the woman’s usual GP or to a health professional with mental health training and expertise. Obstetricians should make themselves aware of referral options to psychologists, psychiatrists, social workers or services in their local area An EPDS score of 12 or more should be repeated within 2 weeks, and a very high EPDS score should be investigated further as it may suggest a crisis
3	Guidelines for Preventive Activities in General Practice 2016	RACGP *National*	Guideline	Perinatal	Pregnant and postpartum women are considered to be at “increased risk”. *Recurrent screening* may be more useful in people deemed to be at higher risk of depression; recommend *screening opportunistically* Clinicians should maintain a high level of awareness for depressive symptoms in patients at high risk of depression and make appropriate clinical assessments wherever the risk is high	GPs	EPDS	Opportunistic screening recommended; frequency and timing not specified	Not specified
4	Perinatal and Infant Mental Health Model of Care 2015	Department of Health, Western Australia *State*	Consultation	Perinatal	Develop a *screening* and assessment process/schedule, across sectors, at regular time-points during pregnancy, the postnatal period, infancy and early childhood. Promote *universal screening* using reliable and valid tools for early identification of perinatal mental health disorders, parental and infant/child mental health problems, and assessment of family functioning	Clinicians of various professional disciplines: Antenatal (GP, obstetrician, practice nurse/midwife, midwife, mental health nurse, child health nurse, Aboriginal health worker, counsellor, psychologist, volunteer worker) Postnatal (GP, child health nurse, midwife, obstetrician, practice nurse, mental health nurse, Aboriginal health worker, psychologist, counsellor, pharmacist)	EPDS or culturally validated tools (e.g., Kimberley Mums Mood Scale)	Depression/anxiety (EPDS) screen and psycho-social assessment at least twice during pregnancy, in the first and third trimesters CHN home visits to routinely screen for paternal, maternal and infant well-being, a ascertain other important factors in the home environment that may contribute to psychological morbidity, and to determine level of further support required for the family GP visits to routinely screen women/men throughout the first 12 months post-partum to assess physical and mental wellbeing and support healthy lifestyle decisions Regular checks of child and parent health and wellbeing and family functioning across the 12-month postpartum period as demands on parents change over time	Ensure that intervention is implemented as early as possible for babies and children identified at risk, and for their parents Following a comprehensive assessment, care plans are developed in consultation with the woman and her partner/support person and shared with those involved in her care during the perinatal period, allowing for modification over time Care plans and protocols involve assertive monitoring, particularly in the first few weeks after childbirth and early intervention for patients with a history of mental illness, as well as for those identified at significant risk Include in the care plan: treatment for the mental health problem, how frequently during the perinatal period monitoring should occur and the roles of all healthcare professionals, including who is co-ordinating the plan
5	Mental health care in the perinatal period: Australian clinical practice guideline 2017	COPE *National*	Guideline	Perinatal	Use the EPDS to screen women for a possible depressive disorder in the perinatal period	All health professionals providing care to women in the perinatal period	EPDS	Complete the first antenatal screening as early as practical in pregnancy and repeat screening at least once later in pregnancy Complete the first postnatal screening 6–12 weeks after birth and repeat screening at least once in the first postnatal year Repeat the EPDS at any time in pregnancy and in the first postnatal year if clinically indicated	Provide structured psychoeducation to women with symptoms of depression in the perinatal period Arrange further assessment of perinatal woman with an EPDS score of 13 or more. For a woman with an EPDS score between 10 and 12, monitor and repeat the EPDS 2–4 weeks later as her score may increase subsequently Repeat the EPDS at any time in pregnancy and in the first postnatal year if clinically indicated For a woman with a positive score on Question 10 on the EPDS undertake or arrange immediate further assessment and, if there is any disclosure of suicidal ideation, take urgent action in accordance with local protocol/policy
6	Clinical Practice Guidelines, Pregnancy Care 2019	Department of Health, Australia *National*	Guideline	Perinatal	Use the Edinburgh Postnatal Depression Scale (EPDS) to *screen* women for a possible depressive disorder	Health professionals (midwife; GP; obstetrician; Aboriginal and Torres Strait Islander health worker; multicultural health worker)	EPDS	Conduct screening as early as practical in pregnancy and repeat at least once later in pregnancy. Repeat the EPDS at any time in pregnancy if clinically indicated	Identify appropriate health professionals available to provide follow-up care and to assist if there are concerns for the safety of the woman or foetus. Identify other professionals from whom you can seek advice, clinical supervision or support regarding mental health care in the antenatal period If concerned about the woman’s mental health and safety, contact mental health services Arrange further assessment of woman with an EPDS score of 13 or more. For a woman with an EPDS score between 10 and 12, monitor and repeat the EPDS in 4–6 weeks as her score may increase subsequently. Repeat the EPDS at any time in pregnancy if clinically indicated
UNITED STATES									
7	Health Care Guideline: Depression in Primary Care 2016	ICSI *State*	Guideline	Perinatal	Clinicians should *screen* and monitor depression in pregnant and post-partum women. *Routine* maternal *screening* is highly recommended, followed by a clinical interview of those scoring above threshold	Clinicians	EPDS, PHQ-2 or PHQ-9	Clinicians should screen and monitor depression in pregnant and post-partum women As per recommendation for all adults, including pregnant and postpartum women: The optimum interval at which to screen for depression is unknown; more evidence for all populations is needed to identify ideal screening intervals	Routine maternal screening is highly recommended, followed by a clinical interview of those scoring above threshold Clinicians should establish and maintain follow-up with patients
8	Final Recommendation Statement: Depression in Adults: Screening 2016	USPSTF *National*	Recommendation statement	Perinatal	The USPSTF recommends *screening* for depression in the general adult population, including pregnant and postpartum women	Clinicians	EPDS	Not specified for perinatal populations	Screening should be implemented with adequate systems in place to ensure accurate diagnosis, effective treatment, and appropriate follow-up All positive screening results should lead to additional assessment that considers severity of depression and comorbid psychological problems, alternate diagnoses, and medical conditions
9	Screening for Perinatal Depression 2018	ACOG *National*	Committee Opinion	Perinatal	The American College of Obstetricians and Gynecologists (the College) recommends that obstetrician–gynaecologists and other obstetric care providers *screen* patients at least once during the perinatal period for depression and anxiety symptoms using a standardized, validated tool	Obstetrician-gynecologist and other obstetric care providers	Validated tool (e.g., EPDS, PHQ-9)	It is recommended that all obstetrician–gynaecologists and other obstetric care providers complete a full assessment of mood and emotional well-being (including screening for postpartum depression and anxiety with a validated instrument) during the comprehensive postpartum visit for each patient. If a patient is screened for depression and anxiety during pregnancy, additional screening should then occur during the comprehensive postpartum visit	When indicated, health care providers share a role in initiating medical therapy or referring patients to appropriate behavioural health resources, or both
10	Obstetric Care Consensus No. 8: Interpregnancy Care 2019	ACOG and the Society for Maternal–Fetal Medicine *National*	Consensus	Postnatal	*All* women should be *screened* for depression in the postpartum period and then as part of well-woman care during the interpregnancy period. Postpartum depression screening also may occur at the well-child visit with procedures in place to accurately convey the information to the maternal care provider	Health care providers: obstetrician-gynecologists, primary care providers, subspecialists who treat chronic illness, advanced practice professionals and mental health providers	Validated tool (e.g., EPDS, PHQ-9)	In the postpartum period at well-child visits and then during interpregnancy interval	Consider referral to mental health providers Postpartum depression screening also may occur at the well-child visit with procedures in place to accurately convey the information to the maternal care provider
11	Incorporating Recognition and Management of Perinatal Depression into Pediatric Practice 2019	AAP *National*	Policy Statement	Postnatal	Routine screening for postpartum depression should be integrated into well-child visits at 1, 2, 4 and 6 months of age	Paediatric primary care clinicians	EPDS, PHQ-9	Routine screening in which a validated screening tool is used should occur at well-infant visits at 1, 2, 4 and 6 months	When a depression screen result is positive, management will vary according to the degree of concern and need Management of postpartum depression includes demystification, support resources and referrals for the mother (to a mental health professional or the mother’s primary care clinician or obstetrician) As with any mental health crisis in which suicidality is a concern, referral to emergency mental health services (most communities have mental health crisis teams or services) is needed, and the mother should only leave with her support person or under the care of community resources, such as mental health crisis services or emergency medical services
12	Position Statement 49: Perinatal Mental Health 2018	MHA *National*	Position Statement	Perinatal	*Maternal depression screening and intervention* should be fully implemented in obstetrics and paediatrics, in addition to adult preventive care visits Ensure that *screening* is consistent, both during pregnancy and in the postpartum period, and that positive *screens* are followed-up with timely and effective services	Provider in any healthcare setting	PHQ-9, EPDS	Ensure that screening is consistent, both during pregnancy and in the postpartum period	Mental health professionals should be co-located within the settings where screening is performed to provide immediate evaluation, diagnosis and treatment of mothers with positive screening results. Where physical co-location is not feasible, virtual co-location by telehealth is a reasonable alternative, and other innovations, such as use of peer support specialists, primary care-led group-based interventions or use of phone applications for peer support should be tested Screening should be reviewed by the provider for immediate follow up by mental healthcare professionals that are co-located in the screening setting Ensure that **…** positive screens are followed-up with timely and effective services
13	Position Statement: Mental Health During Childbirth and Across the Lifespan 2020	American College of Nurse-Midwives *National*	Position Statement	Perinatal	*All* perinatal clients should be evaluated for depression and other mental health disorders at least twice during pregnancy and at regular intervals postpartum. ACNM recommends *screening* for depressive disorders with a validated tool	Healthcare Providers	Validated tool (none specified)	All perinatal clients should be evaluated for depression and other mental health disorders at least twice during pregnancy and at regular intervals postpartum	Every midwifery practice should have a systematic response to a positive screen or risk assessment, including knowledge of treatment modalities and referral to trained mental health providers
CANADA									
14	Family Physician Guide: for Depression, Anxiety Disorders, Early Psychosis and Substance Use Disorders 2008	Ministry of Health, British Columbia *Province*	Guide	Postnatal	Recommending *universal screening* of all women at the 2-month postpartum visit using the EPDS. The EPDS may also be used in pregnancy to screen for suspected depression	Service provider (GP, OB/GYN, midwife, community health nurse, childbirth education, doula, pregnancy outreach program)	EPDS	At the 2-month postpartum visit using the EPDS. This scale can be readministered at any time within the first 12 months following birth of a baby. The EPDS may also be used in pregnancy to screen for suspected depression	A positive score on item #10 should be taken seriously. Safety of the mother needs to be discussed A marginal screening result is a score of 10 or 11, readminister in 2 weeks Discuss women’s responses, being alert to a mismatch with your clinical impression. The EPDS should never be used in isolation, it should form part of a full and systematic mood assessment of the mother, supporting professional judgement and a clinical review
15	Recommendations on screening for depression in adults 2013	Canadian Task Force on Preventative Health Care *National*	Guideline	Perinatal	For adults in subgroups of the population who may be at increased risk of depression (including perinatal and postpartum status), we recommend *not routinely screening* for depression	Not applicable	Not applicable	Not applicable	Not applicable
16	Best Practice Guidelines for Mental Health Disorders in the Perinatal Period 2014	Perinatal Services BC *Province*	Guideline	Perinatal	Assuming care pathways are established, *screen all* women for perinatal depression	Healthcare providers	EPDS	Screen using the EPDS at least once during pregnancy and once in the postpartum period. Suggested timeframes for administering the EPDS are: 28 to 32 weeks gestation, 6 to 16 weeks postpartum and anytime concerns are identified	EPDS score 9–11; depression possible; support, re-screen in 2–4 weeks, consider referral to primary care provider EPDS score 12–13; fairly high possibility of depression; monitor, support and offer education, refer to primary care provider EPDS score 14 and higher (positive screen); probable depression; diagnostic assessment and treatment by primary care provider and/or specialist Positive score (1, 2, or 3) on question 10 (suicidality risk); immediate discussion required. Refer to primary care provider + mental health specialist or emergency resource for further assessment and intervention as appropriate. Urgency of referral will depend on several factors including: whether there has been a history of suicide attempts, whether symptoms of a psychotic disorder are present, and/or there is concern about harm to the baby
17	Assessment and Interventions for Perinatal Depression 2018	Registered Nurses’ Association of Ontario *Province*	Guideline	Perinatal	The expert panel strongly recommends *routine screening* for perinatal depression for *all* pregnant and postpartum persons up to 1 year following childbirth	Trained nurse or member of the interprofessional team	Valid tool, no specific tool promoted, scales mentioned in Appendix E include EPDS, Beck Depression Inventory, Centre for Epidemiological Studies- depression scale, PHQ-9, Postpartum depression screening scale, Whooley scale	As the findings are not consistent, no recommendations regarding specific frequency and timing can be made	Collaborate with the person to develop a comprehensive person-centred plan of care, including goals, for those with a positive screen or assessment for perinatal depression
18	Postpartum Depression Screening 2019	Alberta Health Services *Province*	Guideline	Postnatal	The EPDS shall be *routinely* offered by the Public Health Nurse to all eligible mothers at the first regular Public Health Well Child Clinic visit (generally at 2 months) and may also be offered any time up to 12 months postpartum as indicated. The public health nurse shall offer *screening* to the eligible mother	Public health nurse	EPDS (English or validated translated version) or alternate questions (if EPDS not available)	At the first regular Public Health Well Child Clinic visit (generally at 2 months) and may also be offered any time up to 12 months postpartum as indicated	EPDS 13–30 (in English): The likelihood of depression is considered high and scores 13 and above require a referral for further assessment EPDS 10–12 (in English): The likelihood of depression is considered moderate (may indicate the presence of symptoms that may be distressing) Offer referral and develop a *Family Support Plan* for EPDS score greater than 12 and refer for care depending on the suicide risk referral flowchart
UNITED KINGDOM									
19	Management of perinatal mood disorders 2012	SIGN *National*	Guideline	Postnatal	*Enquiry about depressive symptoms* should be made, at minimum, on booking in and postnatally at 4 to 6 weeks and 3 to 4 months. *For women regarded to be at high risk* (those with previous or current depressive disorder), enquiry about depressive symptoms should be made at *each contact*	Healthcare professionals (The guideline will be of interest to midwives, health visitors, general practitioners, pharmacists, psychiatric nurses, psychiatrists, obstetricians, neonatologists, paediatricians, clinical psychologists, social workers, public health physicians, users of services, and all other professionals caring for women and their families.)	EPDS or Whooley Questions	On booking in and postnatally at 4–6 weeks and 3–4 months	Where there are concerns about the presence of depression, women should be re-evaluated after 2 weeks. If symptoms persist, or if at initial evaluation there is evidence of severe illness or suicidality, women should be referred to their general practitioner or mental health service for further evaluation
20	Antenatal and Postnatal Mental Health 2018	NICE *National*	Guideline	Perinatal	At a woman’s first contact with primary care or her booking visit and during the early postnatal period, *consider asking depression identification questions* as part of a general discussion about a woman’s mental health and wellbeing and using the EPDS or the PHQ-9 as part of monitoring	Healthcare professional	Two depression identification Questions (Whooley), if positive or any clinical concern then use EPDS or PHQ-9	At all contacts after the first contact with primary care or the booking visit, the health visitor and other healthcare professionals who have regular contact with a woman in pregnancy and the postnatal period (first year after birth), should consider: • asking the 2 depression identification questions and the GAD-2 (see recommendation 5.3.8.4) as part of a general discussion about her mental health and wellbeing and • using the EPDS or the PHQ-9 as part of monitoring	If depression identification questions are positive, consider referral to GP or, if a severe mental health problem suspected, to a mental health professional If a woman responds positively to either of the depression identification questions in recommendation 5.3.8.4, is at risk of developing a mental health problem, or there is clinical concern, consider using the Edinburgh Postnatal Depression Scale (EPDS) or the Patient Health Questionnaire (PHQ-9) as part of a full assessment referring the woman to her GP or, if a severe mental health problem is suspected, to a mental health professional
NEW ZEALAND									
21	Identification of Common Mental Disorders and Management of Depression in Primary Care 2008	Ministry of Health, New Zealand *National*	Guideline	Perinatal	At a pregnant woman’s first contact with primary care, her “booking” visit and 6-week postnatal check, the practitioner should *consider the use of the verbal 2–3 question screening tool* for depression as part of *routine* assessment	Practitioner	Verbal 2–3 question screening tool (Whooley) for depression	At a pregnant woman’s first contact with primary care, at her “booking” visit and 6-week postnatal check, the practitioner should consider the use of verbal 2–3 question screening tools for anxiety and substance abuse as part of routine assessment	If a woman’s response to a verbal 2–3 question screening tool arouses concern about a possible mental disorder (or if other issues do so), she should normally be referred promptly for further clinical assessment by her general practitioner/practice nurse team

*AAP* American Academy of Pediatrics, *ACNM* American College of Nurse Midwives, *ACOG* American College of Obstetitcians and Gynecologists, *BC* British Columbia, *CHN* Child Health Nurse, *COPE* Centre of Perinatal Excellence, *EDS* Edinburgh Depression Scale, *EPDS* Edinburgh Postnatal Depression Scale, *GAD* Generalised Anxiety Disorder, *GP* General Practitioner, *ICSI* Institute for Clinical Systems Improvement, *MHA* Mental Health America, *NSW* New South Wales, *PHQ* Patient Health Questionnaire, *RACGP* The Royal Australian College of General Practitioners, *RANZCOG* The Royal Australian and New Zealand College of Obstetricians and Gynaecologists, *SIGN* Scottish Intercollegiate Guidelines Network, *USPSTF* United States Preventive Services Taskforce

When extracting data relating to the recommendation made, care was taken to extract information specifically pertaining to “screening”, wherever possible. When the term “screening” was not used but aforementioned terminology including but not limited to “assessment” or “enquiry” was used instead, then these were extracted. If a mix of terms was used, then care was taken to extract information pertaining to “screening”, only. Recommendations, including those relating to frequency, timing, follow-up and referral, were extracted verbatim where possible (Table 2). It should be noted that in some instances the extent of data available necessitated that the research team summarise key details during extraction into Table 2, as relevant to the aims of this review.

When extracting information relating to the provider responsible, care was taken to use the exact terminology used in each publication. When the title of the recommendation was targeted towards a specific healthcare profession (e.g. if the recommendations were developed for GPs specifically), then it was assumed that they would be responsible for screening, unless otherwise stated. In the event where extensive follow-up and referral recommendations are made, these are summarised in Table 2. Where any of this information is not reported, “not specified” is stated in Table 2.

## Results

### Search results and screening

**Fig. 1:**
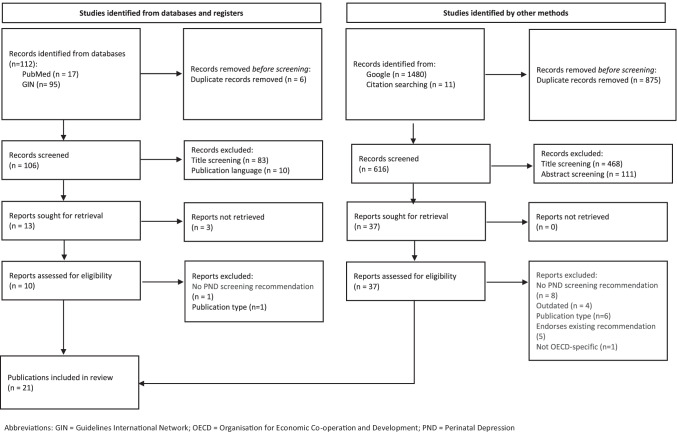
Flow chart of article selection process, adapted from the PRISMA 2020 flow diagram for new systematic reviews (Page et al. 2021) Abbreviations: GIN, Guidelines International Network; OECD, Organisation for Economic Co-operation and Development; PND, perinatal depression

The search yielded 1592 publications, of which 1480 were from Google; 17 were from PubMed; and 95 were from GIN. A further 11 publications were identified during screening and data extraction (citation searching). After duplicate removal, publications were screened based on title, abstract (when available) and then full-text. The screening process yielded 21 included publications (Fig. 1).

### General characteristics

Publications originated from the United States (US) (*n* = 7) (ACOG 2018; American College of Nurse-Midwives 2020; Earls et al. 2019; Mental Health America 2018; Obstetric Care Consensus No. 8: Interpregnancy Care 2019; Siu et al. 2016; Trangle et al. 2016), Australia (*n* = 6) (Australian Government Department of Health 2019; COPE 2017; NSW Department of Health 2009; RACGP 2016; RANZCOG 2015; WA Department of Health 2015), Canada (*n* = 5) (Alberta Health Services 2019; BC Ministry of Health 2008; BC Reproductive Mental Health Program 2014; CTFPHC 2013; Registered Nurses’ Association of Ontario 2018), the United Kingdom (UK) (*n* = 2) (NICE 2018; SIGN 2012) and New Zealand (*n* = 1) (New Zealand Guidelines Group 2008). Eleven (ACOG 2018; Alberta Health Services 2019; American College of Nurse-Midwives 2020; BC Reproductive Mental Health Program 2014; COPE 2017; Earls et al. 2019; Mental Health America 2018; NICE 2018; RANZCOG 2015; Registered Nurses’ Association of Ontario 2018; SIGN 2012) publications addressed perinatal mental health, of which seven were specific to perinatal depression, anxiety or mood disorders (ACOG 2018; Alberta Health Services 2019; BC Reproductive Mental Health Program 2014; Earls et al. 2019; RANZCOG 2015; Registered Nurses’ Association of Ontario 2018; SIGN 2012). Five (BC Ministry of Health 2008; CTFPHC 2013; New Zealand Guidelines Group 2008; Siu et al. 2016; Trangle et al. 2016) publications addressed depression in adults, of which two (CTFPHC 2013; Siu et al. 2016) were specific to depression screening. Two publications (NSW Department of Health 2009; WA Department of Health 2015) addressed family mental health; another two publications (Australian Government Department of Health 2019; Obstetric Care Consensus No. 8: Interpregnancy Care 2019) focused on pregnancy care; and one publication (RACGP 2016) focused on preventative health care in adults. Fourteen publications (ACOG 2018; American College of Nurse-Midwives 2020; Australian Government Department of Health 2019; COPE 2017; CTFPHC 2013; Earls et al. 2019; Mental Health America 2018; New Zealand Guidelines Group 2008; NICE 2018; O'Connor et al. 2016; Obstetric Care Consensus No. 8: Interpregnancy Care 2019; RACGP 2016; RANZCOG 2015; SIGN 2012) were published by national organisations; and seven (Alberta Health Services 2019; BC Ministry of Health 2008; BC Reproductive Mental Health Program 2014; NSW Department of Health 2009; Registered Nurses’ Association of Ontario 2018; Trangle et al. 2016; WA Department of Health 2015) publications were published by state/province-based organisations (Table 2).

### Recommendations

As can be seen in Table 2, there are variations in the terminology used when making PND screening recommendations, which may have implications for how the recommendations are used and implemented. Sixteen publications recommended screening for depression, or perinatal mood disorders or symptoms of these illnesses specifically and often specified a recommended screening tool (ACOG 2018; Alberta Health Services 2019; American College of Nurse-Midwives 2020; Australian Government Department of Health 2019; BC Ministry of Health 2008; BC Reproductive Mental Health Program 2014; COPE 2017; Earls et al. 2019; Mental Health America 2018; Obstetric Care Consensus No. 8: Interpregnancy Care 2019; RACGP 2016; RANZCOG 2015; Registered Nurses’ Association of Ontario 2018; Siu et al. 2016; Trangle et al. 2016; WA Department of Health 2015). Some publications were less directive, in that “screening” was not recommended specifically, rather recommendations that healthcare professionals “enquire about depressive symptoms” (SIGN 2012), “consider asking depression identification questions” (NICE 2018) or “consider the use of the verbal 2–3 question screening tool” (New Zealand Guidelines Group 2008) were made. However, the use of screening tools such as the EPDS was still often suggested. One publication recommended conducting “psychosocial assessment” (NSW Department of Health 2009), recognising that psychosocial assessment includes depression screening or recommending the use of depression screening tools. The Canadian Task Force on Preventive Health Care (CTFPHC) recommended against routine screening, citing a lack of evidence and noting that “undesirable effects probably outweigh the desirable effects” (CTFPHC 2013). This recommendation was graded as “weak” with “very low quality evidence” (CTFPHC 2013). Nonetheless, being “alert to the possibility of depression” was still recommended in this publication (CTFPHC 2013). Among publications that supported screening, some recommended universal screening (BC Ministry of Health 2008; NSW Department of Health 2009; WA Department of Health 2015) and/or screening for “all” women (ACOG 2018; Alberta Health Services 2019; American College of Nurse-Midwives 2020; BC Reproductive Mental Health Program 2014; Registered Nurses’ Association of Ontario 2018), while others recommended opportunistic screening (RACGP 2016). Furthermore, there was a variation as to whether publications recommended screening routinely (Alberta Health Services 2019; Earls et al. 2019; New Zealand Guidelines Group 2008; RANZCOG 2015; Registered Nurses’ Association of Ontario 2018; Trangle et al. 2016) or specified a minimum number of times screening should be offered, for example, at least once (ACOG 2018; Australian Government Department of Health 2019; BC Reproductive Mental Health Program 2014; COPE 2017) or twice (WA Department of Health 2015).

### Screening tools

Eighteen (ACOG 2018; Alberta Health Services 2019; Australian Government Department of Health 2019; BC Ministry of Health 2008; BC Reproductive Mental Health Program 2014; COPE 2017; Earls et al. 2019; Mental Health America 2018; NICE 2018; NSW Department of Health 2009, 2010; Obstetric Care Consensus No. 8: Interpregnancy Care 2019; RACGP 2016; RANZCOG 2015; SIGN 2012; Siu et al. 2016; Trangle et al. 2016; WA Department of Health 2015) publications included the EPDS among the recommended screening tools, including for screening among populations for whom English is not their first language, as it has been translated into other languages (NSW Department of Health 2009). Six (Alberta Health Services 2019; Australian Government Department of Health 2019; BC Reproductive Mental Health Program 2014; COPE 2017; NSW Department of Health 2009; Registered Nurses’ Association of Ontario 2018) publications recommended a translated version of the EPDS when screening perinatal women of culturally and linguistically diverse backgrounds, of which five (Australian Government Department of Health 2019; BC Reproductive Mental Health Program 2014; COPE 2017; NSW Department of Health 2009; Registered Nurses’ Association of Ontario 2018) noted the need for culturally relevant cut-off scores. However, not all translated versions of the EPDS have been validated (Department of Health Government of Western Australia 2006), with Australian publications highlighting the lack of validated screening tools for people from non-English speaking backgrounds (COPE 2017) and recognizing that the translated EPDS may not account for cultural sensitivity and cannot be considered acceptable nor valid for all populations (WA Department of Health 2015).

The PHQ-9 was among the recommended tools in six (ACOG 2018; Earls et al. 2019; Mental Health America 2018; NICE 2018; Obstetric Care Consensus No. 8: Interpregnancy Care 2019; Trangle et al. 2016) publications, of which five were from the US. One publication recommended using the PHQ-9 for screening women from non-English speaking backgrounds (Trangle et al., 2016). Additionally, the PHQ-2 was recommended in two publications (NICE 2018; Trangle et al. 2016).

The New Zealand and UK National Institute for Health and Care Excellence (NICE) publications (New Zealand Guidelines Group 2008; NICE 2018) recommended asking two depression identification questions, first, followed by a screening tool (e.g. EPDS or PHQ-9) if the “woman responds positively” to either question. The Scottish Intercollegiate Guideline Network (SIGN, 2012) publication recommended the use of EPDS or Whooley questions to “aid clinical monitoring and to facilitate discussion of emotional issues”, rather than for screening explicitly, citing a lack of evidence to support the tools’ “sufficient accuracy” in the perinatal period. The Whooley questions, sometimes not referred to by name but described in-text, were identified amongst included publications (New Zealand Guidelines Group 2008; NICE 2018; SIGN 2012).

### Screening timing and frequency

When defining the postpartum period, this was often considered to be up to 1 year postpartum (COPE 2017; NICE 2018). The majority of publications made recommendations across the perinatal period; however, some publications aimed to provide guidance postnatally specifically. For example, publications with a focus on postpartum depression screening, interpregnancy care or perinatal care in paediatric practice were identified (Table 2). Due to their focus on the postnatal period, it was not unexpected that the depression screening recommendations within such publications were often specific to the postnatal period.

The recommendations relating to screening intervals ranged from “as early as practical” (Australian Government Department of Health 2019; COPE 2017; NSW Department of Health 2009), or at “first contact” (New Zealand Guidelines Group 2008; NICE 2018) to 32 weeks (BC Reproductive Mental Health Program 2014) or in the third trimester (WA Department of Health 2015) antenatally and 4 weeks to up to 6 months postnatally (Earls et al. 2019). Some publications did not make specific recommendations for frequency and timing, and there was recognition among included publications that “findings have not been consistent” in regard to this issue (Registered Nurses’ Association of Ontario 2018).

### Provider responsible

It is evident from the data presented in Table 2 that a broad range of healthcare providers are considered responsible for screening. The terms used to describe them included “clinicians” (NSW Department of Health 2009; RANZCOG 2015; Siu et al. 2016; Trangle et al. 2016), “practitioners” (New Zealand Guidelines Group 2008; RANZCOG 2015), “all health professionals providing care to women in the perinatal period” (COPE 2017), “healthcare professionals” (NICE 2018; SIGN 2012), “healthcare providers” (American College of Nurse-Midwives 2020; BC Reproductive Mental Health Program 2014) and “provider in any healthcare setting” (Mental Health America 2018). In some publications, it was implied which “clinicians” or “practitioners” were specifically responsible, for example, midwives, child and family health nurses (NSW Department of Health 2009) or obstetricians and gynaecologists (RANZCOG 2015). There was also consideration for the healthcare settings (e.g. mental health, obstetric, paediatric, primary care, emergency, occupational health settings) in which screening could occur (Mental Health America 2018). Recommendations which were more specific about responsible providers were often published by organisation or bodies specific to the profession. For example, the publications by the Royal Australian College of General Practitioners (2016) and the American Academy of Pediatrics (Earls et al. 2019) discussed screening by general practitioner paediatric primary care clinicians, respectively.

### Referral and follow-up recommendations

The EPDS cut-off scores for further assessment varied across included publications (Alberta Health Services 2019; Australian Government Department of Health 2019; BC Reproductive Mental Health Program 2014; COPE 2017; RANZCOG 2015). For example, a cut-off score of “13 or more” was recommended (Australian Government Department of Health 2019; COPE 2017), as was a cut-off score of “at least 12” (Alberta Health Services 2019). There was recognition that the EPDS may need to be completed multiple times. For example, one publication suggested that a cut-off score of 12 or more warranted monitoring and that the screen be repeated in 2 weeks with a broader recommendation that a “very high EPDS score should be further investigated” (RANZCOG 2015). Some publications made different recommendations for different cut-off scores, whereby an EPDS score of 12 to 13 required monitoring and possible referral, while an EPDS score of 14 or higher required “diagnostic assessment and treatment” (BC Reproductive Mental Health Program 2014).

A broad range of referral recommendations were made, including to various healthcare professionals (BC Reproductive Mental Health Program 2014; Earls et al. 2019; NICE 2018; NSW Department of Health 2009; Obstetric Care Consensus No. 8: Interpregnancy Care 2019; RANZCOG 2015; SIGN 2012), resources (ACOG 2018; BC Reproductive Mental Health Program 2014; Earls et al. 2019), services (Earls et al. 2019; NSW Department of Health 2009; RANZCOG 2015; SIGN 2012), the development of a care or treatment plan (Alberta Health Services 2019; NSW Department of Health 2009; RANZCOG 2015; Registered Nurses’ Association of Ontario 2018; WA Department of Health 2015), treatment (Mental Health America 2018; RACGP 2016) and/or further assessment (Australian Government Department of Health 2019; COPE 2017; New Zealand Guidelines Group 2008; Siu et al. 2016). Healthcare professionals that were specifically mentioned in referral recommendations included GPs (NICE 2018; NSW Department of Health 2009; RANZCOG 2015; SIGN 2012), psychologists (NSW Department of Health 2009; RANZCOG 2015), social workers (RANZCOG 2015) and obstetricians (Earls et al. 2019). Healthcare professionals recommended were also referred to more broadly as “mental health providers” (Obstetric Care Consensus No. 8: Interpregnancy Care 2019) or “mental health professionals” (Earls et al. 2019; NICE 2018), “primary care clinicians” (Earls et al. 2019), or “primary care providers” and “mental health specialists” (BC Reproductive Mental Health Program 2014).

Services recommended included “community adult mental health services” (NSW Department of Health 2009), “services in their local area”(RANZCOG 2015) and “mental health services” (SIGN 2012). Resources recommended included “behavioural health resources” (ACOG 2018), “emergency resource” (BC Reproductive Mental Health Program 2014) as well as “mental health crisis services” and “emergency medical services” (Earls et al. 2019). Publications also often referred to local protocol and policy when suggesting further assessment (Australian Government Department of Health 2019; COPE 2017) and some acknowledged the need to consider depression severity and comorbidities (Siu et al. 2016).

## Discussion

Most publications identified in this systematic review recommended PND screening for all women, routinely. However, the guidance provided varied in relation to the terminology used, timing and frequency of screening, screening tool to be used, the health care provider responsible and the appropriate follow-up and referral pathways. Even though most publications supported the detection of PND, some did not recommend “screening” specifically in their recommendations, acknowledging the limited evidence, especially in relation to the effectiveness of screening, benefit of screening, specifics of timing and frequency of screening and responsibility for screening, as well as a lack of established pathways for post-screening referrals (BC Reproductive Mental Health Program 2014; Registered Nurses’ Association of Ontario 2018; SIGN 2012; Siu et al. 2016).

This systematic review identified a lack of consensus in relation to the recommended timing and frequency of PND screening. Screening at first contact during pregnancy was often recommended (COPE 2017; Department of Health and Human Services 2019; Registered Nurses’ Association of Ontario 2018) as the appropriate timing for antenatal screening. The lack of consistency in timing of screening recommendations postnatally may be a reflection of the lack of consensus regarding the definition of the postnatal period (Wisner et al. 2010). For example, in the context of depressive disorders, the ICD-11 and DSM-5 (American Psychiatric Association 2013; World Health Organisation 2020) define the postnatal period as 4 or 6 weeks postpartum, whereas published literature alludes to 12 months postpartum (Woolhouse et al., 2015). Conversely, the term “perinatal” is defined as the perinatal period as commencing at “22 completed weeks of gestation” and ending at “seven completed days after birth” by the World Health Organisation and has been used to describe foetal and neonatal outcomes, rather than maternal outcomes (World Health Organisation 2006), further adding to the confusion in terminology. This may be further complicated by studies that have investigated the longer-term prevalence of depression, such as at 3 and 5 years postpartum, that have reported similar prevalence rates of depression to those reported in studies up to 12 months postpartum (Najman et al. 2000; Wang et al. 2011; Woolhouse et al. 2015). Guidance on PND screening after the 12-month postpartum period was not identified, raising the question as to when the postpartum period ends and the maternal period begins and indicating a need for standardizing terminology, as well as exploring the utility of screening tools in the longer term. Furthermore, historically, terms including “woman” and “mother” are used when referring to people experiencing PND; however, it is important to recognise that those experiencing PND may not always identify as “woman”, “she” or “mother” (Registered Nurses’ Association of Ontario 2018). Awareness of and sensitivity when using appropriate pronouns and terms, which may include “person” or “parent”, are particularly important. Future research into tailored care and appropriate terminology is needed to ensure inclusivity and reduce barriers to healthcare.

Not surprisingly, most of the included publications recommended the use of the EPDS for PND screening due to its effectiveness, validity or reliability, availability in languages other than English as well as ease of use and brevity. While the EPDS is a simple screening tool, healthcare professionals need to be trained in its use to be able to score and interpret responses (Thombs et al. 2014) as well as make decisions regarding optimal cut-off points based on the aim of screening, so as to maximise sensitivity, specificity or both (Levis et al. 2020). Furthermore, its reliability and validity as well as its appropriateness when used with culturally and linguistically diverse populations need to be considered. For example, cultural concerns have been reported in relation to using the EPDS among First Nations mothers; however, its cultural validity may vary across countries, demonstrating the need for further research regarding its use with diverse populations (Chan et al. 2021). Nonetheless, the EPDS has been used by a broad range of healthcare professionals, including general practitioners, obstetricians, paediatricians, midwives, nurses and multicultural health workers to screen for PND across diverse populations and, it has been found to be acceptable in a variety of contexts (El-Den et al. 2015).

This systematic review identified heterogeneity in recommendations pertaining to which healthcare provider was responsible for screening, specifically. Screening should be provided in universal, accessible care settings, such as primary care (Howard & Khalifeh 2020), which encompasses a range of healthcare settings, including but not limited to family physicians/GP practices and community pharmacy and PND screening by a broad range of healthcare professionals who interact with women in the perinatal period, including but not limited to paediatricians and nurses, is acceptable (El-Den et al. 2015). Hence, if screening and follow-up are to be integrated in “all medical settings that encounter perinatal women” (Flynn et al. 2006), then healthcare professionals working in these settings require sufficient training to explain the rationale for screening, conduct screening, interpret and explain screening scores and triage for timely diagnosis and management (A Buist et al. 2007; El-Den et al. 2015). While the role of some specific healthcare professionals, such as pharmacists, was not specifically mentioned in regard to screening, with terminology alluding to “all” and “primary” healthcare professionals within included publications, perhaps their role in screening is implied in some recommendations. However, healthcare professionals frequently report that formal perinatal mental health education is insufficient in curricula (Elkhodr et al. 2018; Legere et al. 2017; Sambrook Smith et al. 2019). Hence, there is a need to develop, evaluate and integrate purpose-designed education with input from mental health professionals and consumers. Preliminary research among trained pharmacy students indicates that they were capable of assessing for suicide risk (82.4%) and referring to appropriate healthcare professionals (88.2%), as demonstrated during role-plays with simulated patients exhibiting symptoms of postnatal depression (S. El-Den et al. 2018b), suggesting that integrating perinatal mental health education in primary healthcare curricula may allow future healthcare professionals to contribute to early detection and referral.

The CTFPHC (2013) explicitly recommended against routine screening for PND; however, the publication did note that “clinicians should be alert to the possibility of depression”, especially for those who might be at high risk. This recommendation against screening was based on a review published in 2012 which included 5 studies, all of which were conducted by the same author(s) in rural regions of Japan among elderly populations, as no other studies were considered eligible for inclusion (Keshavarz et al., 2012). While, a more recent review of 14 studies, with broader inclusion criteria, reported that PND screening was beneficial “in terms of increasing referral to, and engagement with, appropriate support services”, it also acknowledged the need for further research exploring long-term outcomes (Reilly et al. 2020). Furthermore, previous reviews have demonstrated that there is a lack of evidence to demonstrate that screening for PND benefits perinatal women, highlighting the need for high-quality RCTs in this area (Thombs et al. 2014). Moreover, a systematic review exploring the cost-effectiveness of interventions for PND and anxiety could not draw conclusions due to the heterogeneity across studies, but reported that interventions incorporating identification and treatment were likely to be cost-effective (Camacho and Shields 2018). The cost of perinatal mental health problems also needs to be considered in such evaluations, with estimates indicating that the estimated costs of perinatal mental health problems are £8.1 billion per year’s birth in the UK (Bauer et al. 2014) and $877 million per annum for depression and anxiety in Australia, indicating a need to develop a “high quality and comprehensive screening program…in collaboration with health professionals and consumers” (PwC Consulting Australia 2019).

Despite evidence of screening effectiveness in terms of reduced depression risk and increased service use (O'Connor et al. 2016; Reilly et al. 2020), the impact of PND screening on improving detection and management of depression is likely to be low if appropriate referral and follow-up pathways are not integrated for diagnosis and treatment (Hazell Raine et al. 2020; Siu et al. 2016). Among some included publications, screening was often recommended under the assumption that follow-up, care and referral pathways are established and available (BC Reproductive Mental Health Program 2014). For example, the USPSTF recommended screening be “implemented with adequate systems in place to ensure accurate diagnosis, effective treatment and appropriate follow-up” (Siu et al. 2016). However, it is unclear how to determine whether such assumptions have been met and who is responsible to ensure these pathways do exist, making decision-making difficult for the individual healthcare professional. Furthermore, even in high income countries there is often a lack of mental health services and workforce shortages that render referrals even more difficult. For example, women in approximately half of the UK do not have access to specialist perinatal mental health services (Bauer et al. 2014), and there is a “critical shortage” of perinatal mental health service and assessment pathways in Australia (Hazell Raine et al. 2020). Decisions to recommend against screening or to refuse/withdraw funding for screening may be due to the lack of established post-screening pathways, but may also be due to a lack of evidence to demonstrate the sustainability of screening outcomes and potential long-term benefits of screening among children (Hazell Raine et al. 2020). Hence, there is a need for high-quality, well-designed studies exploring the effectiveness of PND screening, by a range of healthcare professionals across healthcare settings, to determine pathways for referral, diagnosis, treatment and recovery as well as the long-term impact and cost-effectiveness of PND screening.

Finally, it is important to also recognize that, in some instances, differences in recommendations pertaining to PND screening may be necessary. There are sometimes vast differences across countries, in terms of availability of trained healthcare workforce, settings and pathways for screening as well as prevalence of and risk factors for depression; and these differences can also exist within a country. Hence, there is often a need for PND screening and treatment recommendations that are specific to certain locations and populations (Hansotte et al., 2017). Nonetheless, it is evident from the current review that further clarity is needed for individual healthcare professionals to be able to use these recommendations to guide their clinical practice and for the development of a comprehensive approach to the early detection of PND based on current evidence. In line with findings of a review, exploring postnatal depression screening and treatment for low-income women within Western countries, the current review also highlights the importance of considering how recommendations will actually be implemented, especially among vulnerable populations (Hansotte et al., 2017). Further research focusing on implementation of screening recommendations across diverse settings and populations can facilitate the development and updating of guidelines, policies and recommendations in this area.

### Strengths and limitations

Findings should be interpreted while considering the potential limitations of this systematic review. Due to the heterogeneity in publication types and lack of consistency in terminology used across publications, it was not possible to conduct a quality assessment. Another potential limitation is that that this systematic review only identified guidelines and recommendations from five out of 37 countries that were members of the OECD at the time of searching, which may be due to only including publications published in the English language which were available online at the time searching, screening and extraction were conducted. It is possible that other guidelines and recommendations have become available online or have been published or updated, since the current review was conducted and that these were not captured. It is also possible that webpages were updated during the months of screening and extraction, and this may have influenced the publications that were ultimately included in this systematic review; however, this is the nature of the online environment from which included publications were identified. Furthermore, while every effort was made to include any publication issued by a body within an OECD member country that included a PND screening recommendation, adherence to the inclusion and exclusion criteria by research team members may have resulted in the exclusion of publications at the title or abstract stage which did not make clear their intention to make recommendations within the body of the text. Future research exploring recommendations published in other languages, through other mediums and by other authors, as well as those issued by countries that are not members of the OECD is warranted.

As stated in “Methods”, where possible data extraction is verbatim; however, when needed, the authors of this review had  to summarise data from included publications to ensure data extraction was relevant to the review aims, as well as, for the purpose of clarity, consistency and conciseness in Table 2. While care and effort were taken to ensure no assumptions were made on behalf of an individual publication’s authors, it is possible that differences in terminology and understanding, as well as, publication structure and flow resulted in errors in data extraction. Nonetheless, to the authors’ knowledge, the strength of this review is that it is the first to aim to systematically identify and compare PND screening recommendations, paving the way for further research and inquiry in this area.

## Conclusion

This systematic review identified 21 publications from OECD member countries, of which the majority supported PND screening. Recommendations relating to the timing, frequency, screening tool and healthcare provider responsible varied. There is a need to establish and evaluate referral and follow-up pathways. While evidence of the effectiveness of screening is limited, given the high prevalence of PND and the potential negative consequences of undetected and unmanaged depression on parents and children, ensuring PND is detected, referred and managed early is essential. Nonetheless, screening and associated referral services need to be integrated into the broader health system in a manner that allows for coordinated care by multiple healthcare professionals who are involved in the care of perinatal women. Future research exploring the outcomes of screening by a broad range of healthcare professionals and evaluating perinatal mental health education in healthcare curricula is warranted.
